# Relationship between Isokinetic Knee Strength and Speed, Agility, and Explosive Power in Elite Soccer Players

**DOI:** 10.3390/ijerph19020671

**Published:** 2022-01-07

**Authors:** Jaroslaw Kabacinski, Piotr M. Szozda, Krzysztof Mackala, Michal Murawa, Agata Rzepnicka, Piotr Szewczyk, Lechoslaw B. Dworak

**Affiliations:** 1 Department of Biomechanics, Poznan University of Physical Education, Królowej Jadwigi 27/39, 61-871 Poznan, Poland; murawa@awf.poznan.pl; 2 Myofascial Therapy Center “FizjoMED +”, Ul. Ziębicka 24/1, 60-164 Poznan, Poland; szozda@fizjomedplus.pl; 3 Department of Track and Field, University School of Physical Education, Ul. Paderewskiego 35, 51-612 Wroclaw, Poland; krzysztof.mackala@awf.wroc.pl; 4 Public Medical Center ‘Doktor Krasicki’ in Gdynia, Ul. Zakręt od Oksywia 3, 81-244 Gdynia, Poland; rzepnicka.agata@gmail.com; 5 Faculty of Health Sciences, Calisia University, Ul. Nowy Świat 4, 62-800 Kalisz, Poland; p.szewczyk@akademiakaliska.edu.pl (P.S.); l.dworak@akademiakaliska.edu.pl (L.B.D.)

**Keywords:** isokinetic, knee extensors and flexors, soccer, vertical jump, speed, agility

## Abstract

The aim of this study was to determine the relationship between the isokinetic characteristics of knee extensors and flexors with selected motor abilities: Speed, agility, and explosive power of lower extremities of professional football players in the preparation period of a yearly training cycle. Twenty-one players (age: 24.5 ± 3.9 years; body mass: 76.7 ± 4.7 kg and body height: 183.5 ± 5.5 cm) playing in the highest Polish soccer league participated in the study. The isokinetic concentric torque of the knee extensors and flexors was measured at 300°/s, 180°/s, and 60°/s velocities. Sprint performance was assessed in the 30 m sprint test (standing start). The forward, lateral, and backward movements were assessed using the T-Test of agility. Explosive power was quantified by performing the squat jump (SJ) and countermovement (CMJ), using the force platform. Due to sport-specific demands of soccer activities measured in this experiment, the relationships between peak torque (PT) and the 30 m sprint, T-Test of agility, and power of vertical jumps (SJ and CMJ) were low or medium at speeds of 60°/s and 300°/s. One of the main reasons for the lack of high dependence of the above-mentioned factors are that the measurements were performed during the initial training period where the level of individual abilities is at a low level. Additionally, this experiment may also indicate that the measurement of isokinetic knee flexion and extension peak is effective when performed at the correct angular velocity in relation to the evaluation of the intended motion structure.

## 1. Introduction

Soccer is all about intermittent high-intensity exercises. Strength, speed, and anaerobic power, especially of the lower limbs, strongly affect other motor activities [1,2]. It also includes multi-faceted and continuous changes of direction, which are characterized by dynamic actions related to forward and backward running and lateral movements [3]. These actions occur when the intensity of movement changes, along with jumping, kicking, and tackling movements [4,5]. Additionally, a soccer game requires technical skill and tactical awareness [6], of which the implementation and performance depend largely on the player position, and the league that the player is in [7]. According to Krustrup [8], sprints and high-intensity efforts, including dribbling, jumps, and kicking, account for about 10–15% of the total activity on the field. However, such a low percentage of covered running distance seems to be the most critical and decisive movement structure influencing the performance (results) of the soccer game [9].

All of these factors can be key determinants of a successful soccer performance [9], and therefore it may be important not only to assess basic qualities of these skills but, above all, to rationally apply training to develop these abilities. In this connection, regular monitoring of the most important training data of motor fitness, especially during the preparation period, is important for the structural improvement, progression of performance, and above all, it allows one to reduce the occurrence of injuries [10]. In addition, monitoring the parameters of motor fitness and related technical skills will allow the identification of the strengths and weaknesses of players in terms of their motor functioning and their direct participation in the game [2,3]. An efficient training process leads to an increase in motor abilities and technical skills, and thus the achievement of specific adaptive changes in the body usually takes place based on increasing the player’s strength potential. According to Lehnert et al. [11], greater strength is associated with enhanced force-time characteristics (e.g., rate of force development and capability of producing external mechanical power), which is a requirement of sprinting, change of direction, jumping, and soccer-specific skills. Therefore, the isokinetic assessment of muscle strength (predicting the state of muscle strength) is of particular importance in football games [11,12,13,14].

One of the most reliable and commonly used methods of evaluating the level of muscle strength is isokinetic dynamometry [15]. In isokinetic testing [16], lower concentric angular velocities are most often used for measuring maximum strength and higher concentric angular velocities (with a higher number of repetitions) for determining stamina. Wrigley [17] claimed that isokinetic tests of the knee flexors and extensors are reliable and sensitive enough to investigate the variation in the strength of soccer players. Since the knee joint is one of the major contributors to sprinting and vertical jumping, several studies have attempted to correlate these tests, but with different results [6,9,18,19,20]. Differences in outcomes may be due to a number of differences, such as the angular velocity of the joints and position of the participants, affecting the length and rate of muscle contraction, and the characteristics of the participants [21]. The assessment of knee flexor and extensor strength levels could help develop an effective training program focused on improving speed and agility skills in soccer. In addition, it will also be a qualitative indicator in lower limb power development, based on the application of vertical jumps (SJ or CMJ). All these elements will allow one to determine the appropriate load on the players’ knee joints and, above all, to prevent injuries. Therefore, this study aimed to determine the relationship between isokinetic knee flexors and extensors torque reached at three angular velocities and in functional sport-specific tests such as sprinting, agility, and power of vertical jumps performance in male professional soccer players.

## 2. Material and Methods

### 2.1. Study Design

A randomized controlled trial design was used to determine the effect of knee flexors and extensors on soccer-related performance variables, such as linear speed, agility, and explosive power, of lower extremities in professional male soccer players. The measurement of selected motor ability—the linear speed at 30 m, T-Test of Agility, power output, and jump height during the squat jump (SJ) and counter-movement jump (CMJ)—was taken during the first week of the special preparation period for the autumn round of the yearly soccer training program. The isokinetic concentric torque of the knee extensors and flexors was measured for both lower extremities separately at 300°/s (10 repetitions), 180°/s (7 repetitions), and 60°/s (5 repetitions) velocities. The protocol included a 15 min warm-up. The soccer players were prohibited to engage in high-intensity physical activity 48 h prior to the tests. All tests were performed within 4 days: T-Test of Agility and 30 m (first day), vertical jumps (second day), and the isokinetic test (third and fourth days).

### 2.2. Participants

Twenty-one players (age: 24.5 ± 3.9 years; body mass: 76.7 ± 4.7 kg and body height: 183.5 ± 5.5 cm), all members of a professional KS Warta Poznan soccer team playing in the First Division of Polish Soccer League, participated in the study. On average, participants took part in 5–6 soccer training sessions and played one competitive 90-min match per week. All participants had an average 11.7 ± 3.9 years of soccer training experience. The inclusion criterion was no history of musculoskeletal or cardiorespiratory complaints in the past three months. Before conducting the study, all participants were informed of the experiment’s risks and signed an informed consent form at the first session of the experiment. Ethical approval for this study was obtained from The Bioethical Commission of the Poznan University of Medical Sciences (No 203/08).

### 2.3. Isokinetic Torque Measurement

For each lower extremity, the isokinetic torque of the knee extensors and flexors was measured using the Biodex System 3 dynamometer (Biodex Medical Systems Inc., New York, NY, USA). The laboratory was air-conditioned, and the room temperature was maintained between 22 and 24 °C. Prior to testing, the players completed a supervised general 15 min warm-up that included stationary cycling for 5 min at a self-regulated moderate intensity, 5 min of dynamic stretching exercises for the major muscle groups, and a few vertical jumps with a progressive increase in intensity of jump performance. Isokinetic measurements were carried out for the concentric contraction at the three angular velocities: 300°/s, 180°/s, and 60°/s. The players sat upright on an adjustable dynamometer seat, and each subject’s trunk, pelvis, and thighs were strapped to prevent additional body movement. The range of motion (RoM) was set from 0° (knee extension) to 90° (knee flexion). The knee joint rotation axis was identified through the lateral femoral condyle and aligned with the motor axis. Gravity error torque was recorded for every subject. The first lower extremity test was assigned randomly for each subject. There was no verbal encouragement during testing repetitions. The testing protocol on the dynamometer involved one warm-up set at the same velocity that followed in the testing set and one testing set of concentric muscle actions. In the warm-up set, the players performed 5–10 concentric flexion/extension actions with progressive increases in the effort to reach a maximum. The participants performed a set of 10 maximal repetitions at the 300°/s velocity. The break between different testing velocities was 3 min. After the break, players performed 7 maximal repetitions at the 180°/s velocity, then after another break, ended the experiment with 5 maximal repetitions at the 60°/s velocity. When testing of one side was completed, a 2 min’ break followed, during which the dynamometer setting was changed to accommodate for the opposite lower extremity. The variables collected from isokinetic testing were the absolute peak torque, time of peak torque, and power of the knee extensors and flexors.

### 2.4. Soccer Motor Ability Measurement

Countermovement and Squat Jump:

The soccer players started by performing two vertical jumping tests (SJ and CMJ) to measure the average explosive power of lower extremities. The jumps were assessed using the piezoelectric force platform 1000 Hz (Kistler type 9261A, Winterthur, Switzerland) and Multi Vertical Jump 1.0 software. Before the main jumping performance, they did a short 5-min warm-up, ending with a demonstration of the jumps and performing practice jumps. Each player performed three trials of SJ with their arms on the hips and three trials of CMJ with an arm swing. The best results of jump height and power for SJ and CMJ were taken for further analysis. The rest interval between trials was 30 s. A three-minute recovery was designated between both jumps (SJ and CMJ).

**Figure 1:**
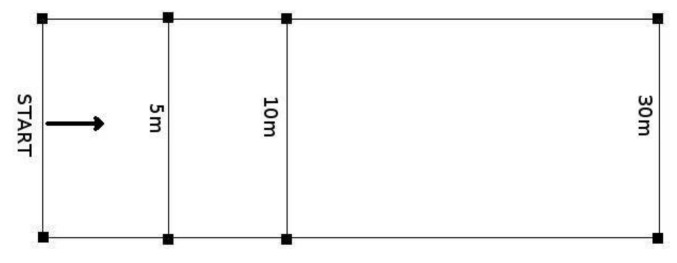
The layout of the of 30-m dash sprint.

Linear sprint and agility performance:

Before a 30-m dash sprint test, participants performed a standard 15-min running warm-up consisting of easy jogging, stretching, skips, and at the end, the performance of two progressive (until 90% of maximal effort) 30 m sprints. Then, two trials were completed, with the best time used for analysis. Between the trials, a 3-min break was enforced to allow full recovery. A Timing System (STS—Poland) was set up with four photocells to measure the total and intermediate times at the start line, 5 m, 10 m, and one at the end of the 30 m (Figure 1). In addition, the time needed to cover the first 5 and 10 m was recorded—in order to analyze the characteristics of acceleration. The starting position involved standing with the front foot positioned behind the first pair of STS gates.

**Figure 2:**
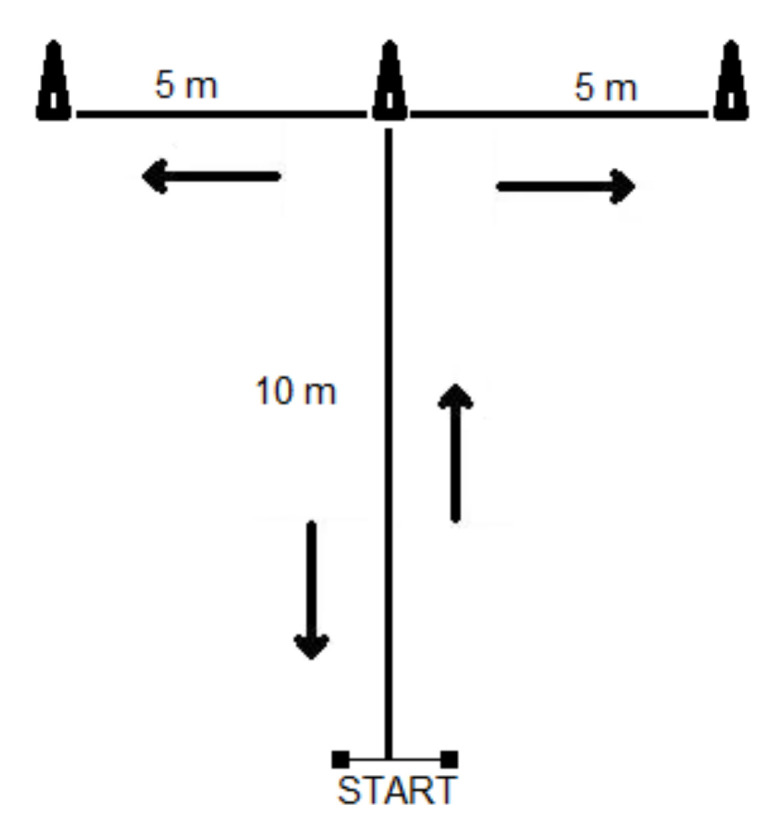
The layout of the T-Test of Agility modified from Semenick [22].

After 10 min of rest, the agility test was performed. The forward, lateral, and backward movements were assessed using the T-Test of Agility. Subjects began with both feet behind the starting line then sprinted 10-m straight, then turned to the right and shuffled 5-m and touched the cone with the right hand. Players then shuffled to the left 10-m and touched a cone at the end with the left hand. They then shuffled right to the center 5-m, touched the cone, and then ran backwards 10-m, passing the finishing line. The total distance of running was 40 m. The T-Test of Agility performance time was measured using two photocells in the Timing System (STS—Poland) at the start finish line. The time was measured when soccer players passed the photocell, and it stopped immediately when the participants passed photocells again at the finish line. Two trials were executed, and the best time was chosen for the final analysis. The break between attempts was 3 min. Before testing, subjects were allowed to perform one trial at their own pace (Figure 2).

### 2.5. Statistical Analysis

Statistical analysis was performed using the Statistica 13.1 program (StatSoft, Inc., Tulsa, OK, USA). The normal distribution of data was verified by the Shapiro–Wilk test. The paired sample *t*-test and Wilcoxon signed-rank test were used to compare the isokinetic variables between the lower extremities. Relationships were determined using the Pearson and Spearman correlations. The level of statistical significance was set at *p* < 0.05.

## 3. Results

Table 1 shows the means and standard deviations of the peak torque, time to peak torque, and average power for 300°/s, 180°/s, and 60°/s as well as *p* for the comparisons between the lower extremities.

**Figure 3:**
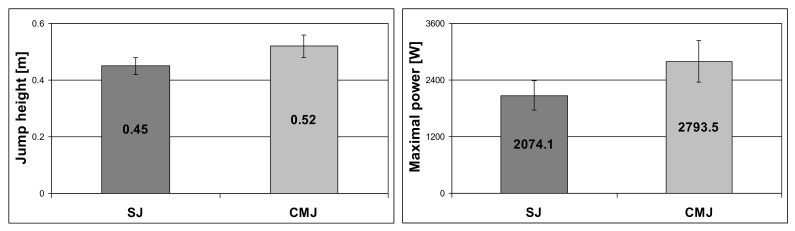
Results of the jump height and maximal power during the SJ and CMJ.

Results of the Shapiro–Wilk test (*p* > 0.05) indicated that data of the time to peak torque for 300°/s (flexors), 180°/s (left extensors), and 60°/s (right extensors and flexors) as well as average power for 60°/s (right flexors) was not normally distributed. Our analysis showed a significant difference in the values of time to peak torque for flexors between the lower extremities (*p* = 0.006). Results of the jump height and maximal power during SJ and CMJ are presented in Figure 3.

**Figure 4:**
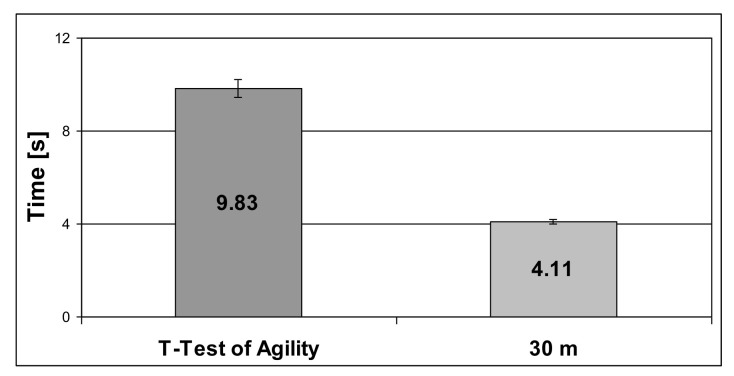
Results of the time for T-Test of Agility and time for 30 m speed test.

Results of the time for the T-Test of Agility and time for the 30 m speed test are shown in Figure 4.

Correlation coefficients for the relationships between jump height, maximal power and peak torque, time to peak torque, and average power for the knee extensors as well as between jump height, maximal power and time for the T-Test of Agility, and time for the 30 m sprint are presented in Table 2.

Results of the Pearson correlation showed a significant relationship between the jump height for SJ and the time for 30 m (*p* = 0.031; moderate correlation) and between the maximal power for CMJ and the peak torque for 180°/s (*p* = 0.030; moderate correlation).

Results of the Pearson correlation revealed a significant relationship between the time for the T-Test of Agility and flexor peak torque for 300°/s (*p* = 0.017; moderate correlation) and 60°/s (*p* = 0.009; moderate correlation), Table 3.

## 4. Discussion

This study investigated the relationship between isokinetic leg strength and sprint and agility performance in a professional KS Warta Poznan soccer team, playing in the First Division of the Polish Soccer League. One of the major findings of this experiment was that when assessed for strength at different velocities (300°/s and 60°/s), a moderate relationship was found only between peak isokinetic knee flexion torque and the T-Test of Agility test (r = 0.513 and r = 0.557, respectively). Furthermore, no dependence was found between peak isokinetic extension torque in the T-Test of Agility test performance and both extension and flexion in the 30 m sprint. Similar information to our results was provided by Cometti et al. [9], Tatlıcıoğlu et al. [19], and Yılmaz et al. 2019 [6], although the research was conducted at slightly different angular velocities: 300°/s and 120°/s, 300°/s and 60°/s, 180°/s and 240°/s, respectively. This study shows none or low to moderate correlations. There are probably several reasons for this, but two or three are the most relevant. An important factor differentiating the achieved results is the level of the studied players, amateur, semi-pro, and pro [6,23,24,25,26], and their experience: Juniors or seniors [11,24,25]. In addition, taking into account the sports level of soccer players [6] and other studies showed that the force coefficient decreases with increasing angular velocity. This statement could be based on the shortest load-range observed at higher velocities in isokinetic conditions.

The strong relationship between flexors’ peak torque and agility highlights the importance of hamstrings in soccer agility-related tasks. Hamstrings are an important biarticular muscle group that concentrically extends the hip, flexes the knee, and participates in tibial rotation as well as eccentrically controlling knee extension. Elevated demands are also placed on the hamstring muscles as one of the main synergists of the gluteus maximus (GM), in cases where there is reduced strength or activation of the GM muscles. Consequently, this synergistic dominance of the hamstring muscles exposes them to a higher occurrence of injuries. High hamstring strength was also highlighted as one of the most common predictors of agility performance [27].

Additionally, the abovementioned results concern the running forms executed by soccer players—linear speed and agility, where there is the ability to stop at a certain speed, change direction (COD), and restart [28,29,30,31]. The two motor structures differ significantly in execution, which explains the magnitude of the relationship with the isokinetic strength of the knee flexors and extensors. In addition, it seems reasonable not to compare players’ results with the results of athletes from other disciplines, such as basketball or volleyball. However, in the process of assessing speed and agility, the same tests can be used for all team games. A review of the literature in this area cannot fully confirm the dependence of the level of isokinetic strength of the knee flexors and extensors in basketball and American football [32,33,34], basketball [35,36], volleyball [37,38], or handball [39]. This could be due to the application of different movement structures in individual team games, the somatic build of players, and, above all, the motor requirements of a given game.

Some studies have shown that there may be associations between the isokinetic torque of knee flexion/extension and other motor structures found in soccer. Our study is focused on the ability to generate explosive power via vertical jumps [21,40,41,42] and the power of various types of ball performance kicking [43,44]. According to these statements, our results reported no significant relationship between knee extensors’ peak torque and jump height and maximal power for SJ and CMJ. The moderate correlation (r = 0.474) appeared only between the maximal power of CMJ and knee extensors’ peak torque for 180°/s. A big surprise was that the results showed no relationship between the jump height and peak torque or average power generated during the performance of these jumps. A moderate relationship (r = −0.473) was found between SJ and the 30 m sprint. These data confirm the results of other experiments, which showed the opposite results—from low or medium to high. For example, Blackburn and Morrisey [45] noticed moderate to high correlations between the mentioned parameters. Contrary to these results, Kovaleski et al. [46] presented only low correlations between the power peak (PT) of knee extensors measured at a velocity of 60°/s as well as PT during leg and single-leg vertical jump performance. In turn, Dauty et al. [47] reported a high correlation of the peak moment of force generated by the extensors of the knee joint at a velocity of 180°/s and the height of the vertical jump: SJ = 0.51; CMJ = 0.65. Similar observations were made by Malloiu et al. [48] in Greek soccer players, who found average and high (0.39–0.78) correlations between the peak moment of force generated by the extensors of the knee joint at a given speed of 60°/s and 180°/s and the height of SJ and CMJ.

As noted in the analysis of this research and comparative data obtained from the quoted literature, there are many opposite outcomes regarding any relationship between isokinetic knee flexor and extensor muscles’ peak torque at different angular velocities and functional performance, such as sprints, agility, and vertical jumps in soccer. Several factors may cause these contradictory results; however, a few of them may be significant. It is generally known that the quadriceps muscle (rectus femoris), significantly involved in the take-off phase in a vertical jump [26,49,50], is a multi-joint closed-motion kinematic chain. On the contrary, extension and flexion of the knee joint is a single-joint movement in an open kinematic chain. Similarly, they respond to sprinting and maneuvering to change direction in agility. These functional differences were made clear in a study conducted by Lehenart et al. [26]. He confirmed that the results of isokinetic muscle strength measurement of the knee joint, and in particular the extensors, provide only partial information about the level of dynamics of the performance of motor structures in soccer. This was also confirmed by Tatlıcıoğlu [19], who claims that there is no inconsistency in terms of movement patterns between the tests; isokinetic testing, sprint testing, and pro-agility are all open kinetic chain movement patterns. Several authors [6,9,19,26,47,48] reported that there were factors related to the specificity of the isokinetic testing protocol, mainly including position, muscle contraction type, and testing angular velocities. Contradictorily to these statements, the latter factor showed a vast spread, mainly above 60°/s, where the measurements were made at the angular velocities of 150, 180, 220, 250, 300, 360, and even 500°/s. There are also noticeable differences regarding age, gender [51,52], training experience, and level [6,24] of sport specificity [26,53]. With a varying degree of impacts, all these factors affect the result, and thus their interpretation in terms of practical use in sports training, not only in soccer. The results of this experiment should be judged against its limitations. The study’s main limitation was only one testing session at the beginning of the preparatory period. The results would probably be slightly different if the test was repeated after some of the training period, e.g., 6–8 weeks. However, different results could have arisen if speed, agility, or plyometric training had been applied at this time. Dividing players into positions could also introduce a different picture of the results obtained, but due to the limited number of players tested, the results could be unreliable. The absence of a control group, e.g., lower-level soccer players, would also show a different view of the results obtained. Testing both the absolute peak torque and relative peak torque could also contribute to the achieved results. This is because the results in sprints, agility, and vertical jumps tests are dependent not only on the technique of performing the movement structure but also on the body mass of the athletes in the case of relative peak torque. All these factors should be taken into account in future research. In addition to these limitations, the obtained results introduce significant knowledge regarding diagnostics in soccer, and thus in sport, and may improve motor training.

## 5. Conclusions

The results of this study indicate that the isokinetic strength of the knee flexors and extensors responded differently in elite soccer players. Due to sport-specific demands of soccer activities measured in this experiment, the relationships between PT and the 30 m sprint T-Test of Agility and power of vertical jumps (SJ and CMJ) were low or medium. Therefore, we cannot draw conclusions on the exact cause of the lack of relationship between isokinetic leg strength and the sprint and agility tests in this study. One of the main reasons for the lack of high dependence of the above-mentioned factors is that the measurements were performed during the initial training period where the level of individual abilities is at a low level. Additionally, this experiment may also indicate that measurement of isokinetic knee flexion and extension peak force is effective when performed at the correct angular velocity in relation to the evaluation of the intended motion structure. This perspective may be a good predictor of sprint, agility, and jumping power performance in advanced male soccer players.

The main practical application of this study is to encourage and make coaches aware of the need for continuous monitoring of the level of motor abilities and related football skills. This is an important action to increase the effectiveness of the game performance, and above all, to reduce the risk of injury.

## Figures and Tables

**Table 1:** Mean ± standard deviation of the peak torque, time to peak torque, and average power as well as statistical test *p*-values.

Variable		300°/s		180°/s		60°/s		*p*-Values (L vs. R)		
		L	R	L	R	L	R	300°/s	180°/s	60°/s
Peak torque [N·m]										
	Extensors	138.0 ± 16.9	132.8 ± 13.6	176.1 ± 15.9	172.0 ± 13.3	245.0 ± 26.9	250.1 ± 25.5	0.179	0.437	0.484
	Flexors	87.1 ± 14.3	89.0 ± 18.2	110.8 ± 15.6	113.5 ± 18.5	144.4 ± 19.0	151.9 ± 29.0	0.679	0.382	0.478
Time to peak torque [ms]										
	Extensors	141.9 ± 16.6	139.5 ± 20.4	203.8 ± 43.7	200.0 ± 39.1	491.0 ± 147.1	513.3 ± 109.4	0.692	0.768	0.368
	Flexors	153.3 ± 27.4	164.3 ± 51.2	204.3 ± 45.3	201.0 ± 49.8	322.4 ± 87.8	384.3 ± 123.4	0.523	0.922	0.006 *
Average power [W]										
	Extensors	276.9 ± 41.6	266.2 ± 36.0	284.6 ± 29.5	275.9 ± 31.2	165.0 ± 21.6	169.9 ± 15.6	0.195	0.493	0.280
	Flexors	165.6 ± 38.3	161.6 ± 44.1	178.7 ± 30.8	182.9 ± 32.9	100.0 ± 17.2	105.6 ± 14.7	0.597	0.597	0.111

Note: L—left lower extremity; R—right lower extremity; *—significant difference (*p* < 0.05; Wilcoxon test).

**Table 2:** Correlation coefficients for the relationships between jump height, maximal power vs. peak torque, time to peak torque, and average power for the knee extensors as well as jump height, maximal power vs. time for T-Test of Agility, and time for 30 m sprint.

Variable.	Peak Torque			Time to Peak Torque			Average Power			Time	
	300°/s	180°/s	60°/s	300°/s	180°/s	60°/s	300°/s	180°/s	60°/s	T-Test of Agility	30 m
Jump height for SJ	0.170	0.166	−0.061	0.202	−0.057	0.016	0.058	0.182	−0.155	−0.146	−0.473 *
Jump height for CMJ	0.222	0.249	−0.109	0.225	−0.169	−0.154	0.074	0.062	−0.109	−0.009	−0.399
Maximal power for SJ	0.103	0.269	0.263	0.215	−0.128	0.263	0.060	0.153	0.125	0.268	−0.368
Maximal power for CMJ	0.267	0.474 *	0.335	0.051	0.080	0.034	0.175	0.370	0.335	0.202	−0.321

Note: SJ—squat jump; CMJ—counter-movement jump; *—significant correlation (*p* < 0.05) for the Pearson test.

**Table 3:** Correlation coefficients for the relationships between time for T-Test of Agility, time for 30 m and peak torque, time to peak torque, average power.

Variable	Peak Torque			Time to Peak Torque			Average Power		
	300°/s	180°/s	60°/s	300°/s	180°/s	60°/s	300°/s	180°/s	60°/s
	Extensors								
T-test agility time	0.345	0.325	0.205	−0.177	−0.275	−0.144	0.303	0.037	0.030
30 m time	−0.204	−0.133	0.044	−0.213	−0.113	−0.315	−0.235	−0.243	0.003
	Flexors								
T-test agility time	0.513 *	0.404	0.557 *	−0.171	−0.202	0.090	0.414	0.142	0.112
30 m time	0.069	0.033	−0.104	0.154	0.003	−0.254	−0.021	−0.180	−0.023

Note: *—significant correlation (*p* < 0.05) for the Pearson test.

## Data Availability

Data supporting reported results can be found at https://www.aszozda@fizjomedplus.pl (accessed on 15 December 2021).
